# Links Between Iron and Lipids: Implications in Some Major Human Diseases

**DOI:** 10.3390/ph11040113

**Published:** 2018-10-22

**Authors:** Stephanie Rockfield, Ravneet Chhabra, Michelle Robertson, Nabila Rehman, Richa Bisht, Meera Nanjundan

**Affiliations:** Department of Cell Biology, Microbiology and Molecular Biology, University of South Florida, Tampa, FL 336200, USA; srockfie@mail.usf.edu (S.R.); chhabra@mail.usf.edu (R.C.); mrobertson1@mail.usf.edu (M.R.); nabila1@mail.usf.edu (N.R.); richabisht@mail.usf.edu (R.B.)

**Keywords:** iron, lipid, obesity, cancer, neurodegeneration, iron chelation, phlebotomy

## Abstract

Maintenance of iron homeostasis is critical to cellular health as both its excess and insufficiency are detrimental. Likewise, lipids, which are essential components of cellular membranes and signaling mediators, must also be tightly regulated to hinder disease progression. Recent research, using a myriad of model organisms, as well as data from clinical studies, has revealed links between these two metabolic pathways, but the mechanisms behind these interactions and the role these have in the progression of human diseases remains unclear. In this review, we summarize literature describing cross-talk between iron and lipid pathways, including alterations in cholesterol, sphingolipid, and lipid droplet metabolism in response to changes in iron levels. We discuss human diseases correlating with both iron and lipid alterations, including neurodegenerative disorders, and the available evidence regarding the potential mechanisms underlying how iron may promote disease pathogenesis. Finally, we review research regarding iron reduction techniques and their therapeutic potential in treating patients with these debilitating conditions. We propose that iron-mediated alterations in lipid metabolic pathways are involved in the progression of these diseases, but further research is direly needed to elucidate the mechanisms involved.

## 1. Introduction

Iron, one of the most essential elements in the human body and indispensable for life, exists in complex forms, including (a) the iron storage complex in which iron is trapped (i.e., hemosiderin), (b) heme containing proteins (i.e., hemoglobin), (c) heme-containing enzymes, (d) transferrin (i.e., holo-transferrin), and (e) the ferritin complex (comprised of 4500 Fe(III) molecules in a complex with ferritin heavy and light chains) [1]. This metal is essential for cellular processes, including metabolic reactions, oxygen transport via hemoglobin, and DNA synthesis [2]. An average adult has 3–5 g of iron in their body [3], while only 1–2 mg of iron is normally absorbed in the intestinal tract, which would then be available for body-wide circulation [4]. Free iron also exists intracellularly in the labile iron pool (LIP) and leads to the production of reactive oxygen species (ROS) via the reaction of hydrogen peroxide (H_2_O_2_) with Fe(II), a process known as the Fenton reaction [5,6]:

Fe(II) + H_2_O_2_+ H^+^ → Fe(III) + [^•^OH] +H_2_O. (1)

Since the body has no mechanism to eliminate excess iron (other than conditions like pregnancy, menstruation, and blood-letting [3]), iron levels must be appropriately maintained to hinder the potentially toxic effects if present in excess [7]. On the other hand, insufficient quantities of iron also leads to detrimental cellular processes [7]. Normal body serum iron levels range from 9–27 µM; however, acute toxicity will be observed in excess of 45 µM. This can result in death if >160 µM, which can be induced by taking iron supplements [3]. On the other hand, chronic iron overload will arise in response to sublethal doses over extended periods of time (i.e., blood transfusions) leading to development of diseases, including cancer [3]. Indeed, the carcinogenic effects of excessive iron have been well established [8]. In this regard, it is interesting that medical conditions, such as hereditary hemochromatosis and β-thalassemia, are associated with an increased risk of developing cancer [3].

Specific mechanisms, such as iron absorption, iron recycling, and iron mobilization, are in place to regulate iron content at both a cellular and systemic level [1]; for comprehensive reviews, see [2,9]. Briefly, uptake of iron, either as transferrin-bound iron (TBI, holo-transferrin bound Fe(III)) or as non-transferrin bound iron (NTBI, Fe(II)), is mediated, respectively, via the transferrin receptor (CD71) and by solute carrier family 39 member 8 (SLC39A8/ZIP8) or solute carrier family 39 member 14 (SLC39A14/ZIP14) [10,11,12]. For uptake of NTBI in liver enterocytes, Fe(III) is oxidized to Fe(II) by duodenal cytochrome b (DCYTB) before being imported into the cell by the divalent metal transporter 1 (DMT1) [13]. After TBI is endocytosed, Fe(III) is released from transferrin and then reduced to Fe(II) by the ferrireductase STEAP3 (six-transmembrane epithelial antigen of prostate 3) [14] prior to its release from the endosome via the DMT1 channel. Cytosolic iron may then (a) remain available for use in the LIP, (b) be transported to mitochondria to generate iron-sulfur (Fe-S) clusters, or (c) be stored within the ferritin complex (a process mediated by poly(RC) binding protein 1 (PCBP1)) [9,15]. Iron is released from the ferritin complex via the action of nuclear receptor coactivator 4 (NCOA4), which is involved in autophagy-mediated degradation of these iron complexes [16,17]. With regards to iron export, ferroportin (FPN1), the only known iron exporter [12], is tightly regulated by hepcidin (HAMP), a protein hormone that is secreted in a controlled manner from the hepatic tissue [12]. The exported iron (in the Fe(II) form) is then oxidized via hephaestin (HEPH) to Fe(III) [18].

Increased circulating transferrin saturation leads to elevated NTBI, which is deposited primarily into the heart, pancreas, liver, and brain [19,20,21,22,23]. Such iron deposits can be observed via transmission electron microscopy (TEM) and are present prior to the development of iron overload symptoms [3]. Under these conditions, ROS accumulates—which can then mediate damage to proteins, lipids, nucleic acids, and other cellular components [3,24,25]. In addition, elevated ROS can induce a ferroptotic response, which is characterized by accumulation of lipid peroxides [15,26]. Activation of ferroptosis promotes cell death in various pathological conditions, such as diffuse large B-cell lymphoma, acute kidney failure, chromophobe kidney cancer, and periventricular leukomalacia [27]. As described later in Section 3, dysregulated iron levels have also been implicated in the development of neurodegenerative disorders, including Alzheimer’s disease, Parkinson’s disease, and Amyotrophic lateral sclerosis [28], as well as in cancer [8].

In addition to systemic iron overload, localized increases in this metal can give rise to conditions, such as endometriosis, a benign gynecological disorder characterized by the presence of endometriotic cysts, which contain old blood components (including heme and its breakdown products) [29]. This source of redox active iron present within these cysts or that arising from follicular fluid, retrograde menstrual effluent, and the process of ovulation have been proposed to contribute to ovarian cancer risk [30,31,32]. Consumption of red meat as a source of dietary iron may also contribute to development of other cancers, namely colorectal cancers [33].

**Figure 1:**
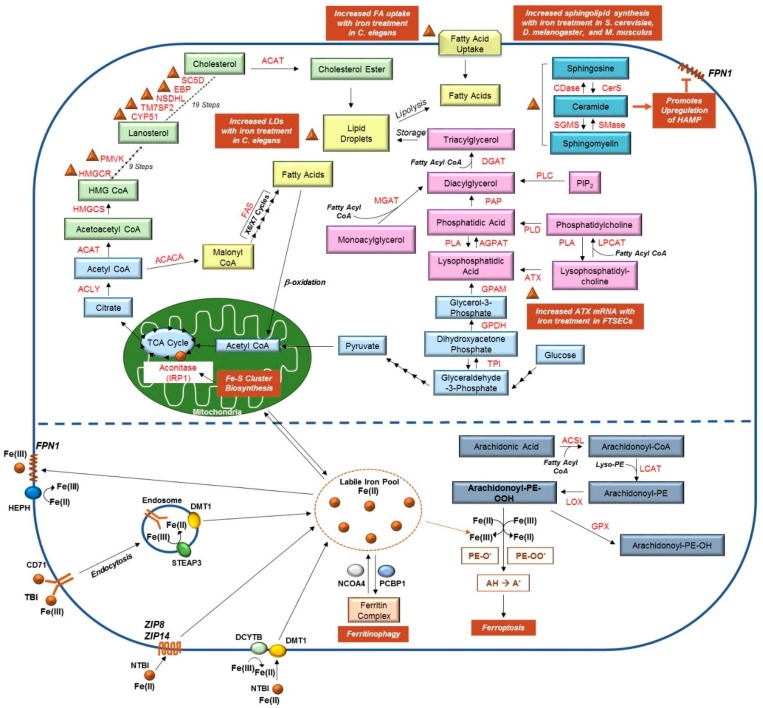
**Links between Lipid and Iron Metabolic Pathways**. (**Top**) Pyruvate, a product of glycolysis, is converted to acetyl-CoA in the mitochondria. Acetyl-CoA feeds into the Krebs (TCA) cycle, illustrated in light blue, to generate citrate; the conversion of citrate to isocitrate is mediated by the enzyme aconitase and requires binding to Fe-S clusters (as indicated by the brown circle). Citrate can also be transported from the mitochondria to the cytosolic compartment where it is used to generate acetyl-CoA; this molecule can then feed into either the cholesterol biosynthetic pathway (green) or the fatty acid synthesis pathway (yellow). Elevated liver iron concentrations correlated with increased mRNA expression of several genes involved in cholesterol biosynthesis (namely, HMGCR, PMVK, CYP51, TM7SF2, NSDHL, EBP, and SC5D), as indicated with brown triangles (see Section 2.1). Exogenous fatty acids can also be imported into the cell; together, fatty acids, triacylglycerides, and cholesterol esters are essential components of lipid droplets. As detailed in Section 2.4, iron can promote both fatty acid import and lipid droplet formation. Synthesis of triacylglycerides, as well as phospholipids (and their modification), are presented in pink, whereas the sphingolipid metabolic pathway is displayed in dark blue. Iron can promote the production of ceramide (indicated with a brown triangle), and in turn induces HAMP expression, which negatively regulates FPN1 (refer to Section 2.2). Furthermore, our own unpublished work suggests iron treatment in human fallopian tube secretory epithelial cells (FTSECs) increases mRNA expression of autotaxin (ATX), which is involved in generating lysophosphatidic acid (see Section 2.5). (**Bottom**) TBI can be imported via endocytosis by binding to CD71; Fe(III) is then converted to Fe(II) by STEAP3 prior to being transported from the endosomal compartment to the cytosolic LIP by DMT1. Alternatively, NTBI can be imported into cells by DMT1 (following conversion of Fe(III) to Fe(II) by DCYTB), ZIP8, or ZIP14 [37]. From the LIP, iron may be (a) transported to the mitochondria for use in Fe-S cluster generation, (b) loaded to ferritin by PCBP1, or (c) used by the cell for other cellular processes. Iron can also be released from the ferritin complex via NCOA4-mediated ferritinophagy. Increased iron levels in the cell can promote the formation of lipid peroxides, a process critical for ferroptosis (shown in grey). Please see [2,38,39,40,41,42,43,44,45] for comprehensive reviews of these pathways and the contributing enzymes.

Similar to iron, lipid levels must be regulated in an appropriate manner to ensure cellular homeostasis. Lipids are a critical source of cellular energy and also have roles as signaling metabolites [34]. When these are in excess, they are stored within lipid droplets to hinder the detrimental effects of lipotoxicity [35]. On the other hand, when lipids are depleted, cellular biosynthetic pathways are activated to generate these macromolecules [35]. Within the body, lipids are primarily stored in adipose tissue, an organ that is also involved in endocrine signaling to regulate energy balance and insulin resistance [36]. Deregulated lipid biosynthetic and catabolic pathways may therefore interfere with crucial biological processes, ultimately producing deleterious effects and potentially causing serious medical issues. Herein, we present a review of the literature pertaining to altered lipid metabolism in response to dysregulated iron pathways. We discuss associations between iron and lipid alterations derived from model organisms (cell lines, *Saccharomyces cerevisiae*, *Caenorhabditis elegans*, *Mus musculus,* and *Drosophila melanogaster*) and patient specimens. As shown in Figure 1, key elements of cholesterol and lipid biosynthesis, iron metabolism, and ferroptosis are summarized, particularly focusing on the interconnections between these pathways, as identified in the studies presented in this review.

## 2. Interactions between Iron and Lipids in Model Systems

### 2.1. Iron and Cholesterol

Cell membranes are comprised of not only protein, but also lipids and cholesterol, which play important roles in cell signaling and maintenance of cell structure [46]. Similar to iron, excess levels of cholesterol can also elicit a toxic effect by elevating oxidative stress responses [47]. Specifically, hepatic iron levels were correlated with increased cholesterol content, which was associated with elevated mRNA levels of seven key enzymes involved in the cholesterol biosynthetic pathway: 3-hydroxy-3-methylglutarate-CoA reductase (HMGCR), lanosterol-14α demethylase (CYP51), ∆14-sterol reductase (TM7SF2), sterol-4α-carboxylate-3-dehydrogenase (NSDHL), cholestenol-∆-isomerase (EBP), phosphomevalonate kinase (PMVK), and lathosterol oxidase (SC5D) [47]. The authors of this work propose that these changes could contribute to the development of fatty liver disease [47].

Cholesterol is also found in lipoprotein particles along with apolipoproteins, such as apolipoprotein E (ApoE), a major brain Apo, which is critical for learning, memory, and brain repair [48]. In Alzheimer’s disease, current evidence implicates impaired levels of ApoE4 (which correlates with neurodegeneration while also being able to bind to metals, such as iron) in the sequestration of iron to amyloid-β deposits [48]. Although elevated levels of iron contribute to increased ApoE mRNA and protein expression, the secretion of this apolipoprotein was reduced [49]. In addition to altered ApoE levels, patients with Alzheimer’s disease have increased ferritin levels in their cerebrospinal fluid (CSF), a marker of brain iron content [50,51,52]. Interestingly, patients with elevated ApoE4 (specifically, the ε4 variant) have >20% increase in CSF ferritin correlating with increased kinetics of cellular degeneration in the hippocampus, as well as with cognitive decline [50,51,52].

The effects of ApoE extend beyond its role in neurodegenerative diseases. For example, ApoE is proposed to protect against NASH (non-alcoholic steatohepatitis) as ApoE knockout mice were characterized by hepatosteatosis [53]. A link to iron was identified in a recent SILAC proteomic study in which adipocytes were treated with ferric ammonium citrate (FAC, a source of NTBI) resulting in an 11-fold increase in ApoE (amongst two other markers), although ApoE secretion was reduced by >55% [53]. Further studies are needed to elucidate the mechanism underlying increased ApoE expression in spite of its reduced secretion.

### 2.2. Iron and Sphingolipids

Like cholesterol, sphingolipids (i.e., sphingomyelin, ceramide, and sphingosine amongst others) are essential membrane and signaling components [54]. The initial link between iron and sphingolipid regulation in eukaryotic systems was derived from *S. cerevisiae* in which iron-induced toxicity was correlated with increased synthesis of sphingolipids [55]. More recently, this finding has been extended to *D. melanogaster* and *M. musculus*, as well as in mammalian cell lines [56,57] in which iron-induced toxicity was mediated by a deficiency in frataxin, a key modulator of iron-sulfur cluster biogenesis, also lacking in patients with Friedreich’s ataxia [58].

Intriguingly, iron uptake via CD71 is increased following cellular treatment with C2-ceramide, a sphingolipid involved in lipid signaling in bovine aortic endothelial cells (BAECs), and could be reversed with an iron chelator (deferoxamine (DFO)) or with an antibody targeting endocytosis of CD71 [59]. C2-ceramide treatment in human hepatocellular carcinoma (HepG2) cells was found to transcriptionally upregulate HAMP mRNA via the JAK/STAT3 signaling cascade [60]. In another report, loss of sphingomyelin in murine lymphoma cells (WR19L) hindered clathrin-mediated endocytosis of CD71 whereas overexpression of sphingomyelin synthase, as well as presentation of exogenous sphingomyelin increased transferrin uptake [61]. Whether alterations in other elements of iron signaling are induced in response to ceramide and other sphingolipids has yet to be determined.

### 2.3. Iron-Sulfur Cluster and Lipids

Iron-sulfur cluster containing proteins play key roles in the Krebs (TCA) cycle (i.e., aconitase) [62] and the electron transport chain (i.e., complex I) [63], which contribute to ATP production. Iron-sulfur clusters are also needed for regulation of enzymes involved in key cellular processes (i.e., DNA polymerase, base excision repair) in addition to iron-sensing molecules (i.e., iron response proteins IRPs) [62]. In human embryonic kidney cells (HEK293) overexpressing a dominant negative form of ISCU (an iron-sulfur cluster assembly enzyme), a 10-fold increase in citrate levels (as a result of a deficiency in aconitase activity) was noted; this citrate was redirected for its use in fatty acid biosynthesis, which increased lipid droplet formation [64,65]. The mitochondrial phospholipid, cardiolipin, is also involved in multiple processes for generating cellular energy by regulating activities of protein complexes involved in the electron transport chain and mitochondrial membrane dynamics [66]. Using the yeast model, researchers identified that deficiency in cardiolipin synthase (crd∆) increased expression of the iron regulon (iron uptake genes for mitochondria) and biogenesis of iron-sulfur clusters in the mitochondria and their export to the cytosol [67]. Additional investigations into cardiolipin regulation needs to be addressed to have an improved understanding of iron-sulfur cluster biogenesis.

### 2.4. Iron, Lipid Droplets, and Leptin

According to the World Health Organization (WHO), obesity (defined as a body-mass index (BMI) ≥ 30) has increased 3-fold over the past 40-decades throughout the world [68]. Additionally, patients who are obese (characterized by a gain in adipose tissues (including visceral and subcutaneous) [69]) are at an increased risk of developing co-morbidities, including cancer [70]. Adipocytes, the major component of these tissues, contain ~100 µm large-sized lipid droplets, composed of triacylglycerides and cholesterol esters [71]. In *C. elegans*, iron supplementation significantly increased the abundance and size of lipid droplets [72]. Specifically, iron treatment in this model organism increased the expression of sgk-1 (an ortholog for mammalian glucocorticoid-induced kinase), which was found to increase expression of acs20 (mammalian homolog, FATP1/4) involved in fatty acid import and thus transport to lipid droplets while simultaneously promoting iron storage in ferritin [72].

Leptin is an adipokine that is produced by adipocytes to regulate hunger [73]. In HuH7, a human hepatoma cell line, leptin treatment resulted in increased HAMP mRNA, which was regulated by the JAK2/STAT3 signaling cascade [74]. Additionally, using mice deficient in leptin (ob/ob), leptin treatment increased both plasma HAMP levels and liver HAMP mRNA, which were associated with an increase in liver iron levels [75]. Interestingly, in mice lacking mediators important for iron efflux (hephaestin and ceruloplasmin), leptin levels were reduced [76]. Furthermore, C57BL/6 mice, fed a high-fat diet, showed increased leptin levels and increased liver HAMP mRNA associated with increased liver iron [77]. Whether leptin alters other elements of iron signaling has yet to be determined.

### 2.5. Iron, LPP1, and Other Enzymes Involved in Lysophospholipid Metabolism

Recent work has identified that overexpression of LIPIN1, an enzyme involved in the conversion of phosphatidic acid (PA) to diacylglycerol (DAG), can reduce iron levels in human hepatic cancer cells (BEL7402) [78]; this phenomenon appeared to be mediated by FPN1, which was increased upon LIPIN1 expression [78]. On the other hand, from our own work (*unpublished results*, *Rockfield and Nanjundan*), we have identified that addition of exogenous NTBI iron (presented as FAC) to transformed gynecological cell lines induced autotaxin (ATX) mRNA. ATX, an adipokine, catalyzes the conversion from lysophosphatidylcholine (LPC) to lysophosphatidic acid (LPA) and is noted to be altered in multiple cancer types, including non-small cell lung cancer, glioblastoma multiforme, melanoma, thyroid cancer, follicular lymphoma, ovarian cancer, hepatocellular carcinoma, breast cancer, and colon cancer [79,80]. To our knowledge, the only other reported link between ATX/LPA and iron is in the H9c2 cardiomyoblast cells; ATX overexpression protected these cells from ferroptotic cell death (an iron-dependent cellular response) by reducing the levels of intracellular ROS [81]. Further work must be performed to improve our understanding of these initial findings.

### 2.6. Iron and Fatty Acid Metabolism

Cancer cells are described to be “addicted” to iron [8]; indeed, their increased proliferative capacity is negatively regulated upon cellular treatment with iron chelators [82]. Links between iron and lipid pathways in cancer are only beginning to come to the forefront. Recent work using a systems biological approach (using existing microarray datasets followed by data mining approaches) implicates associations between the iron pathway and fatty acid synthesis and regulation in high-grade serous epithelial ovarian carcinomas [83]. Specifically, peroxisome proliferator-activated receptor gamma (PPARG), sterol regulatory element binding transcription factor 1 (SREBF1), ATP citrate lyase (ACLY), fatty acid synthase (FAS), acyl-CoA synthetase long-chain (ACSLx)), fatty acid desaturation (fatty acid desaturase 2 (FADS2), stearoyl-CoA desaturase (SCD), elongation of very long chain fatty acid elongase 2 (ELOVL2), elongation of very long chain fatty elongase 5 (ELOVL5)), and glycerolipid metabolic pathways (1-acylglycerol-3-phosphate O-acyltransferase (AGPATx), DGAT1, LIPIN1, LIPIN2, glycerol kinase (GK), glycerol-3-phosphate-acyltransferase (GPAM)) were perturbed along with iron-related genes (iron-ion binding, as well as intracellular iron regulation) [83]. The functional outcomes of these initial associations must be further investigated.

### 2.7. Ferroptosis and Lipids

Lipid peroxides are a form of ROS, which serve as signaling molecules that can alter the properties of cell membranes, lipid interactions, and protein functions. In addition, these ROS promote cellular apoptosis, including iron-dependent ferroptotic cell death [84]. In ferroptosis, recent research has identified alterations in lipid metabolic pathways in addition to the well-established lipid peroxidation [85]. Using retrovirus-generated insertional mutagenesis in KBM7 (haploid chronic myeloid leukemia cells), 9 genes were identified as increased upon ferroptosis induction with multiple small molecule ferroptosis inducers, including ACSL4 (acyl-coA synthetase long-chain family member 4, which produces the arachadonic acid metabolite 5-HETE (5-hydroxyeicosatetraenoic acid)) and LPCAT3 (lysophosphatidylcholine acyl-transferase 3) [86]. Similarly, in a genome wide CRISPR-mediated genetic screen and microarray screen involving ferroptosis resistant cells, ACSL4 was also identified as a regulator of this pathway [87]. Furthermore, ACSL4 mRNA and protein were reduced in ferroptosis-resistant (LnCaP and K562) cells relative to sensitive (HL60 and HepG2) cancer cells [85]. Likewise, in breast cancer cell lines, ACSL4 expression corresponded with ferroptosis sensitivity [87]. When ACSL4 is reduced (via shRNA-mediated knockdown) in HL60 and HepG2 cells, ferroptosis is inhibited; in contrast, when it is overexpressed in LnCaP and K562 cells, ACSL4 promotes ferroptosis [85].

In a ferroptosis mouse model for which glutathione peroxidase 4 (GPX4) is deficient, inhibition of ACSL4 was found to improve tissue health [87]. Furthermore, a novel ferroptosis-inducing compound (CIL56) was identified to be dependent on the activity of acetyl-coA carboxylase 1 (ACC1, the rate-limiting enzyme involved in fatty acid biosynthesis) [86]. Upon knockout of ACC1 via CRISPR-Cas9, a 5-fold increased resistance to ferroptosis was noted in response to CIL56 [86]. In another study, researchers identified LSH (lymphoid specific helicase, a DNA methylase modified, which is part of the SNF2 chromatin remodeling ATPase family) as hindering ferroptosis through its interaction with WD repeat domain 76 (WDR76, via direct promoter binding activity). This in turn corresponded with increased expression of fatty acid desaturases (i.e., FADS2 and FADS5) and was dependent on both iron and lipid peroxidation [88]. In HepG2 and Hep3B liver cancer cells, knocking out the expression of iron sulfur domain 1 (CISD1, localized to the outer mitochondrial membrane) also was found to promote lipid peroxidation and ferroptosis [89]. The clinical utility of such ferroptosis inhibitors could be tested in future work.

## 3. Iron and Lipids: Neurodegenerative Diseases

### 3.1. Brain Iron Localization

Neurodegenerative diseases (i.e., Alzheimer’s, Parkinson’s, and Huntington’s, among others) are considered age-related diseases, in part due to accumulation of iron, and its physiological consequences [90]. Notably, this metal causes inflammation of the brain and thus, its degeneration [90]. Iron response proteins (IRP1 and IRP2) increase amyloid precursor protein (APP) expression, which is the precursor to amyloid-β in Alzheimer’s disease, as well as the expression of α-synuclein, which is a critical component of the Lewy bodies in Parkinson’s disease [90]. The mTOR pathway can regulate expression of CD71, which is responsible for cellular iron uptake [90]; indeed, it has been recently proposed that inhibition of mTOR could reduce iron accumulation and thus, lessen the neurodegenerative effects induced by this metal [90]. Specific brain regions that accumulate iron include the hippocampus [91], the globus pallidus, red nucleus, substantia nigra, dentate nucleus, and caudate-putamen [92], whereas increased iron content in the basal ganglia is a unique feature of a rare brain disease called neurodegeneration with brain iron accumulation (NBIA) [93].

### 3.2. Iron-Mediated Lipid Peroxidation and Ferroptosis

As mentioned earlier, iron participates in the Fenton reaction to generate ROS, which damage lipids via peroxidation [6], a phenomenon observed in neurodegenerative diseases [94]. Indeed, increased redox active iron in certain regions of the brain contributes to the development of neurological diseases, which is associated with programmed cell death [95]. The exact mechanism contributing to this cell death process has been unclear until recently. It is now recognized that iron-dependent cell death pathway, namely ferroptosis, may be involved in the development of such neurodegenerative diseases [95,96].

Lipoxygenases (iron-dependent enzymes) can also promote oxidation of polyunsaturated fatty acids and are localized to the hippocampal region of the brain [97]. One oxidative stress stimulator, namely tert-butylhydroperoxide (t-BHP), promotes cell death via ferroptosis in PC12 cells (a model cell line for neurobiology) by reducing GPX4 protein and glutathione (GSH) levels leading to increased lipid peroxidation [98]. Mitochondrial alterations, including (a) reduced mitochondrial membrane potential, (b) reduced ATP levels, and (c) increased ROS in the mitochondria, were also noted [98]. They further identified that these effects could be reversed upon treatment with ferrostatin or iron chelation with DFO [98]. Neurons in the forebrain (cerebral cortex and hippocampus) are susceptible to ferroptosis [99]. Interestingly, an inducible tissue-specific GPX4 knockout mouse (specifically in forebrain neurons) resulted in massive deficits in cognitive and memory functions concurrently with increased lipid peroxidation, increased MAPK pathway activation, and increased oxidative damage [99]. Furthermore, maintaining these mice on a vitamin E (an antioxidant) deficient diet accelerated the neurodegenerative processes in these mice, whereas treatment with a ferroptosis inhibitor (liproxstatin-1) ameliorated brain functions [99]. In a model of Huntington’s disease using brain slices, ferrostatin-1 was also found to reduce cell death [100]. The clinical application of these ferroptotic inhibitors could be tested in the future.

### 3.3. Iron and the Sphingolipid Pathway

In addition to iron-induced lipid peroxidation, iron can also promote sphingomyelin breakdown via activation of sphingomyelinases, generating the product ceramide (which is involved in mediating the regulated cell death response) [101] that then contributes to neuronal apoptosis, a feature of neurodegenerative diseases. In support, increased levels of sphingomyelin coinciding with reduced ceramide content is associated with neuronal protection and may thus be a targetable pathway (using iron chelators) for treatment [102]. In *D. melanogaster* and *M. musculus*, targeting of frataxin (via mutations or knockout strategies) led to iron-induced toxicity, which was mediated through the sphingolipid/PDK1/MEF2 signaling cascade [56,57]; this was detrimental to the health of these model organisms and recapitulated the neurodegenerative disease, Friedreich’s ataxia [56,57]. Links between iron and the sphingolipid metabolic cascade have been identified in other neurodegenerative diseases, including NBIA; in this disease, sphingolipids were enriched in the compartment with the highest iron levels (i.e., basal ganglia), identified via gene network analyses [93]. The functional contribution of this observation needs to be investigated further in NBIA and in other neurodegenerative diseases.

## 4. Treatments and Concluding Perspectives

Targeting iron and its downstream effectors (i.e., alterations in lipid peroxidation and/or lipid metabolism) would be of high benefit to patients afflicted by detrimental effects of iron accumulation. Methods of iron reduction thus far utilized include iron chelators and the process of bloodletting.

In the case of neurodegenerative diseases, iron treatment with DFO reduced symptoms of Alzheimer’s disease in an amyloid precursor protein (APP) overexpressing transgenic mouse model; specifically, amyloid-β deposits were reduced coinciding with improved cognitive functions [103]. However, in patient studies for a variety of neurological disorders (such as pantothenae kinase-associated neurodegeneration (PKAN), aceruloplasminemia, NBIA, Friedreich’s ataxia, superficial siderosis, Parkinson’s disease, Alzheimer’s disease, and multiple sclerosis), only low to moderate improvement of clinical symptoms was noted in a small proportion of conducted studies, with most observing no improvement [104]. Similarly in cancer, the use of iron chelators, such as DFO, has shown some efficacy in both animal models and clinical studies [105]; for additional iron chelators used in cancer studies, please see citation [105] for more details.

To the best of our knowledge, improvements in health following iron reduction via the process of phlebotomy have been assessed in six independent studies. Patients afflicted with nonalcoholic fatty liver disease (NAFLD) that underwent bloodletting had reduced blood ferritin levels [106]. Administration of phlebotomy in metabolic syndrome (METS) patients reduced blood pressure and heightened insulin sensitivity [107]. Similarly, phlebotomy administration in type II diabetics, characterized by high blood ferritin, had a marked reduction in not only ferritin (at 4-month follow-up), but also in insulin resistance [108]. With respect to cancer patients, there has been a variation in cancer incidence following such bloodletting procedures. In one study, phlebotomy reduced risk of cancer development in 36% of patients [109] while another reported only 4% [110]. Yet another showed a lack of association between iron reduction and overall risk of cancer [111]. Further investigations into implementation of iron reduction therapies can be pursued in future studies.

Although ferroptosis inhibitors have been utilized in *in vivo* animal studies, as well as in vitro studies, novel inhibitors could be designed that could be utilized to treat patients with neurological diseases described herein.
